# Unfractionated heparin displaces sFlt-1 from the placental extracellular matrix

**DOI:** 10.1186/s13293-020-00311-w

**Published:** 2020-06-29

**Authors:** Kyle H. Moore, Heather Chapman, Eric M. George

**Affiliations:** Department of Physiology and Biophysics, University of Mississippi Medical Center, 2500 N State St, Jackson, MS 39216 USA

**Keywords:** sFlt-1, Heparan sulfate, Pregnancy, Heparin

## Abstract

Soluble vascular endothelial growth factor receptor-1 (sFlt-1) is an anti-angiogenic protein which is secreted by numerous cell types and acts as a decoy receptor for the angiogenic protein vascular endothelial growth factor (VEGF). Despite its physiologic importance in maintaining angiogenic balance, excess sFlt-1 levels are associated with the pathogenesis of many diseases, especially those with angiogenic imbalance, endothelial dysfunction, and hypertension. Although sFlt-1 is a soluble protein, it contains a binding site for the extracellular matrix component heparan sulfate. This allows cells to retain and localize sFlt-1 in order to prevent excessive VEGF signaling. During pregnancy, placental syncytiotrophoblasts develop a large extracellular matrix which contains significant amounts of heparan sulfate. Consequently, the placenta becomes a potential storage site for large amounts of sFlt-1 bound to extracellular heparan sulfate. Additionally, it should be noted that sFlt-1 can bind to the anticoagulant unfractionated heparin due to its molecular mimicry to heparan sulfate. However, it remains unknown whether unfractionated heparin can compete with heparan sulfate for binding of localized sFlt-1. In this study, we hypothesized that administration of unfractionated heparin would displace and solubilize placental extracellular matrix(ECM)-bound sFlt-1. If unfractionated heparin can displace this large reservoir of sFlt-1 in the placenta and mobilized it into the maternal circulation, we should be able to observe its effects on maternal angiogenic balance and blood pressure. To test this hypothesis, we utilized in vitro, ex vivo, and in vivo methods. Using the BeWo placental trophoblast cell line, we observed increased sFlt-1 in the media of cells treated with unfractionated heparin compared to controls. The increase in media sFlt-1 was found in conjunction with decreased localized cellular Flt (sFlt-1 and Flt-1) as measured by total cell fluorescence. Similar results were observed using ex vivo placental villous explants treated with unfractionated heparin. Real-time quantitative PCR of the explants showed no change in sFlt-1 or heparanase-1 mRNA expression, eliminating increased production and enzymatic cleavage of heparan sulfate as causes for sFlt-1 media increase. Timed-pregnant rats given a continuous infusion of unfractionated heparin exhibited an increased mean arterial pressure as well as decreased bioavailable VEGF compared to vehicle-treated animals. These data demonstrate that chronic unfractionated heparin treatment is able to displace matrix-bound sFlt-1 into the maternal circulation to such a degree that mean arterial pressure is significantly affected. Here we have shown that the placental ECM is a storage site for large quantities of sFlt-1, and that it should be carefully considered in future studies concerning angiogenic balance in pregnancy.

## Introduction

Soluble fms-like tyrosine kinase-1 (sFlt-1) is a soluble variant of vascular endothelial growth factor receptor-1 (deemed Flt-1) which lacks the transmembrane domain and, thus, its enzymatic capacity [1]. Because its ability to bind pro-angiogenic factors remains intact [2], sFlt-1 behaves as a decoy receptor for vascular endothelial growth factor (VEGF), creating an anti-angiogenic effect. When VEGF is bound to sFlt-1, there is reduced bioavailability to bind to vascular endothelial growth factor receptor-2 (Flk-1), a pro-angiogenic receptor. The subsequent downregulation of angiogenic pathways and lessened production of the vasodilatory agent nitric oxide, which both result primarily through Flk-1 activation [3], can lead to endothelial dysfunction, which has been increasingly associated with hypertension [4]. Under normal physiological conditions, sFlt-1 plays an important role in regulating VEGF activity [5], but when circulating levels exceed its physiological function, pathological consequences may ensue [6]. In addition to binding VEGF, the fourth IgG domains of both Flt-1 and sFlt-1 contain a binding site for heparan sulfate [7], a glycosaminoglycan found in the apical extracellular matrix (ECM) of both the vascular endothelium and the placenta [8, 9]. This allows cells to retain sFlt-1 to control angiogenic balance in their microenvironments. There are two possible molecular sources of excess sFlt-1 in diseased states: (1) increased alternative splicing of the Flt-1 gene to produce the sFlt-1 variants which give rise to soluble receptor forms and (2) proteolytic cleavage of extracellular matrix components which usually sequester and localize sFlt-1, leading to solubilization and mobilization of preexisting sFlt-1 into circulation.

In an intriguing clinical study done by Rosenberg et al., women with uncomplicated pregnancies were prophylactically given heparin throughout gestation. These women saw increases in serum sFlt-1 levels equal to what has been observed in many cases of preeclampsia [10], a pregnancy disorder characterized by systemic maternal endothelial dysfunction leading to complications such as hypertension [11]. Although the exact mechanism in this study was not elucidated, the increases in sFlt-1 were independent of changes in mRNA expression levels [10]. Therefore, it appears that heparin-dependent increases in sFlt-1 were due to non-genomic mechanisms. A probable explanation lies in the fact that heparin and unfractionated heparin, due to their molecular mimicry to heparan sulfate, can also be bound by Flt-1 and sFlt-1 by the same domain that binds heparan sulfate [8]. In pregnancy, heparan sulfate’s binding to sFlt-1 allows the ECM of the endothelium and placenta to act as a large reservoir of sFlt-1 [7]. Because of this, changing the localization of sFlt-1 bound to the endothelial and placental ECM could cause a large increase in circulating sFlt-1 during pregnancy. However, the physiological importance of the ECM in buffering free circulating sFlt-1 levels through its storage and localization of sFlt-1 during pregnancy remains unclear.

Because the study by Rosenberg et al. [10] mentioned above was observational, it is still unclear whether heparin administration during normal pregnancy can cause displacement and release of placental ECM-bound sFlt-1 into the maternal circulation. With the knowledge gained from that study, we proceeded to use unfractionated heparin as a tool to probe the placental ECM for stores of sFlt-1 that could be released from local heparan sulfate and mobilized into the maternal circulation. Here, we hypothesized that by interfering with the normal buffering of free sFlt-1 by the ECM, continuous infusion of unfractionated heparin during pregnancy in rodents would displace bound sFlt-1 from the placental ECM, cause maternal angiogenic imbalance, and ultimately lead to increased maternal blood pressure.

## Materials and methods

### BeWo cell culture

BeWo choriocarcinoma cells (ATCC) were maintained in complete media, DMEM/F12 50/50 (Corning) supplemented with 5% fetal bovine serum (ATCC) and 1% penicillin-streptomycin (GE Healthcare Life Sciences), at 37 °C, 5% CO_2_, and 21% O_2_. Cell displacement experiments were carried out in 24-well plates (Corning) at a seeding density of 1.75 × 10^5^ cells/mL/well and cells were allowed to settle for 24 h before treatment. New media was pre-treated in 8% oxygen for a minimum of 8 h prior to being added to the cells in order for oxygen tension to equilibrate, and the cells were then placed in a nitrogen purged incubator at 8% oxygen for 24 h. An oxygen tension of 8% was selected in order to mimic placental oxygen tension in a normal pregnancy [12]. Additionally, unfractionated heparin (Fresinius Kabi, USA) was added to several wells at a concentration of 0.1, 1.0, or 10 U/mL, while control wells received an equal volume of saline vehicle. After 24 h, media was saved and frozen for later analysis. A sample size of 6 was used for all experimental groups.

### Immunofluorescence

BeWo cells were plated as stated above, with the exception of being in 4-well chambered slides (Bio-Tek) and in room air oxygen (21% O_2_) at a seeding density of 1 × 10^5^/mL/well. After being allowed to settle for 24 h, cells were treated with 10 U/mL unfractionated heparin or saline vehicle as a control. After 2 h of these exposures, the media was aspirated and the slides were washed with iced PBS (GE Healthcare Life Sciences; Logan, UT). Cooled methanol was added to the chamber before incubating for 5 min at 37 °C. The methanol was aspirated and the chamber walls were removed from the slide. The slide was washed in iced PBS and blocked with a 1:10 dilution of normal goat serum in PBS-T (PBS and 0.1% Tween) (Fisher Scientific; Fair Lawn, NJ) solution at 37 °C for 30 min. After aspirating the blocking solution, a rabbit primary antibody for sFlt-1/Flt-1, ab 32152 (Abcam), was combined with the blocking solution at a 1:250 dilution, added to the slide, and incubated for 1 h at 37 °C. The primary antibody solution was aspirated and the slide was washed three times for 5 min in iced PBS. The FITC conjugated goat anti-rabbit secondary antibody, ab6717 (Abcam), was also combined with the serum/PBS-T blocking solution at a dilution of 1:500 and added to the slide for 1 h at 37 °C. The slide was aspirated and washed, followed by the addition of 1:120,000 Hoechst in PBS, which was added for 5 min. After aspirating and three washes in iced PBS, the slide was dried, ProLong Gold AntiFade (Life Technologies; Eugene, OR) was applied, and a coverslip was placed on the slide. An EVOS FL (Life Technologies; Eugene, OR) with fixed gain and brightness setting was used to image the slides and detect the level of background-subtracted normalized fluorescence for a minimum of 12 independent fields as previously described [13].

### Animal welfare

All protocols were approved by the University of Mississippi Medical Center Institutional Animal Care and Use Committee and agreed with the National Institutes of Health Guidelines for Care and Use of Laboratory Animals. Timed pregnant Sprague Dawley rats (Charles River) were received on gestational day 11 (GD 11). Animals were maintained on a 12:12 h light-dark cycle, at constant 23 °C temperature, and given food and water ad libitum.

### Placental villous explant culture

Placental villous explants were obtained and isolated as previously described [14]. Placentas from the vehicle treated animals described below were collected for this ex vivo culture. After tissue excision, placentas were placed in Dulbecco’s phosphate buffered saline (Sigma, St. Louis, Mo). Next, the deciduae were carefully removed and villous bundles of trophospongium and labyrinth were isolated and excised. This process was repeated using multiple placentas until a sufficient number of placental villous explants had been collected. The villous explants were plated in 24-well cell culture plates in Dulbecco’s Modified Eagle’s Media-Ham’s F-12 supplemented with 10% FBS, 100 μg/mL streptomycin, 100 μg/mL penicillin, and 25 μg/mL ascorbic acid. The explants were maintained at 37 °C and at a constant oxygen tension of 21%. Explants were randomly assigned to experimental or control groups, with experimental groups receiving 10 U/mL unfractionated heparin and control groups receiving saline vehicle. Thirty minutes after treatment, the media was removed from the explants and both media and tissue were frozen for further analysis. A sample size of 6 was used for both experimental groups.

### Heparin infusion model

For heparin infusion, osmotic minipumps model 2001 (Alzet) were loaded in a sterile hood on GD14 with either unfractionated heparin or vehicle (sterile saline) and placed intraperitoneally. The effective dose of unfractionated heparin with this regimen was 100 U/kg/day [15–17]. Briefly, animals were anesthetized and maintained with controlled 3% isoflurane (Henry Schein) and a ventral midline incision (~ 2 cm) made to allow for intraperitoneal placement. Carprofen was administered subcutaneously at a dose of 5 mg/kg. Animals were monitored postoperatively for 4 h and were subsequently given half of a 75 mg acetaminophen tablet daily (Bio-Serv). Unfractionated heparin administration began on GD 14, at which point in time the placental masses should have been sufficient to house a substantial amount of bound sFlt-1.

On GD 18, the animals were anesthetized in the same manner as described above and were implanted with carotid catheters, which were exteriorized at the nape of the neck. Animals were monitored postoperatively for 4 h and were given acetaminophen tablets as previously described. The following day, on GD 19, animals were placed in restraint cages and acclimatized for 45 min. Mean pressure was determined over the course of 30 min via direct pressure transducers and a Powerlab receiver (ADI instruments). Animals were then euthanized; fetal and placental weights were measured with some placentas being saved for placental villous explants. Maternal plasma and serum were collected, while organs were preserved by flash freezing in liquid nitrogen. Blood pressure measurement and euthanization were carried out on GD 19 to prevent animals from carrying out their pregnancies to viability. The total number of rats used for in vivo and ex vivo experiments was 17, with *N* = 8–9 per group.

### VEGF and sFlt-1 ELISAs

Released sFlt-1 in media from the cultured cells was measured using a DuoSet ELISA kit (DY321B, R&D Systems; Minneapolis, MN) specific to human Flt-1. Though this antibody can detect both full-length Flt-1 as well as sFlt-1, analyzing media should only detect the soluble forms of the protein. Briefly, a 96-well plate was treated with a capture antibody for 24 h. The plate was washed with the provided buffer and blocked for 1 h with Reagent Diluent. After aspiration, the Flt-1 protein standards and undiluted media samples (in duplicates) were plated and incubated for 2 h. The plate was washed and Flt-1-specific detection antibody was added to the plate for 2 h. The plate was washed, followed by a 20-min incubation with Streptavidin-HRP. The last wash was performed before addition of the color reagent. After 20 min, the Stop solution was added and the plate was read using the Infinite M200 Pro plate reader and associated Magellan software (Tecan; Grodig, Austria). Rat VEGF (DY564, R&D Systems) and sFlt-1 (DY471, R&D Systems) were also measured using DuoSet ELISA kits (R&D Systems; Minneapolis, MN). Although the antibody of the ELISA used for measuring sFlt-1 can detect both full-length Flt-1 as well as sFlt-1, analyzing plasma should only detect the soluble forms (sFlt-1) of the protein. VEGF ELISA intra-assay CV values were 3.7% (1 sample), 5.6% (2 samples), and 2.2% (3 samples), while inter-assay CV values were 7.9% (1 sample), 10% (2 samples), and 4.6% (3 samples). Flt-1 ELISA intra-assay CV values were 7.2% (1 sample), 4.0% (2 samples), and 3.2% (3 samples), while inter-assay CV values were 8.4% (1 sample), 7.2% (2 samples), and 6.3% (3 samples). The protocols for these assays were followed and were the same as that listed above. For plasma free VEGF levels, equal volumes of plasma from each animal were measured in duplicate. For placental VEGF and sFlt-1 measurements, protein was first isolated using a standard RIPA lysing and centrifugation technique. Measurements were then made via ELISA and normalized to the concentration of protein for each individual sample (expressed as pg of VEGF or sFlt-1 per milligram of total protein).

### Quantitative real-time PCR

RNA was isolated using a PureLink RNA Mini Kit (Ambion) and the kit’s protocol was followed. RNA concentration was obtained using a Nanodrop 2000c (Thermo Scientific). According to protocol of the RevertAid First Strand cDNA Synthesis Kit (Thermo scientific), 300 ng of RNA was used to synthesize cDNA. qRT-PCR was performed using a C1000 Touch Thermal Cycler and CFX96 Optics Module Real-Time system head (Bio-Rad). We designed and validated primers specific to full-length rat FLT1, rat sFLT1, and rat heparanase (HPSE). The sequences for these primers are listed below. SYBR green master mix (Thermo scientific), nuclease-free water, and the primers for either FLT1, sFLT1, or HPSE were combined to make master mixes before then being combined with sample cDNA and evaluated using qRT-PCR. Samples were measured in duplicate and normalized to their own β-actin expression (dCT). For each sample’s gene measurement, its expression was then normalized to the control group (ddCT). Data is presented as fold change in expression (2^-^^ddCT^).

Rat FLT1

Forward: GGTGTCTATAGGTGCCGAGC

Reverse: TGGCCCCCTCTTTCAACATC

Rat sFLT1

Forward: TACGTCACAGATGTGCCAAAC

Reverse: GCAGTGCTCACCTCTAACGA

Rat HPSE

Forward: ACAGCACCTACTCACGAAGC

Reverse: GCTGACCAATGTCAGGACCA

### Statistical analysis

All figures display mean data ± standard error. Comparisons between groups were performed by unpaired two-tailed Student’s *t* test (for two group comparisons) or one-way ANOVA (for four group comparisons) with a Tukey’s post hoc test. The significance value cutoff of was *p* < 0.05 and statistical significance is represented by a bar connecting two groups (*p < 0.05, **p < 0.01, ***p < 0.001). All statistical comparisons and graphs were generated with Prism 8 (GraphPad).

## Results

### Heparin displaces sFlt-1 from the extracellular matrix in vitro

**Fig. 1:**
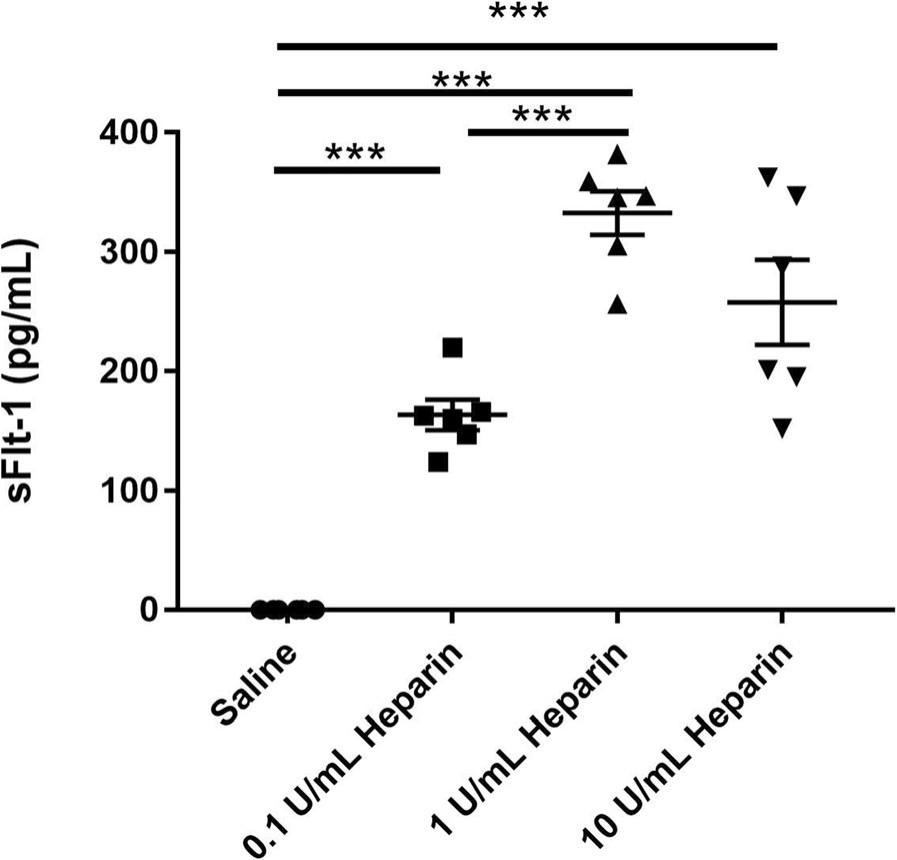
Media sFlt-1 from cultured BeWos. sFlt-1 in the media collected from BeWos cultured in 8% oxygen, treated with either 0.1 U/mL, 1 U/mL, or 10 U/mL unfractionated heparin or saline as a control, as measured by ELISA. *N* = 6 for all groups. Statistical analysis performed using one-way ANOVA with post hoc Tukey’s multiple comparison. Significance is designated by a bar connecting groups (*p < 0.05, **p < 0.01, ***p < 0.001)

Our first aim was to determine whether BeWos treated with unfractionated heparin displace sFlt-1 in a dose-dependent manner. BeWos were cultured in 8% oxygen to mimic the oxygen concentration seen in normal pregnancy and were treated with either 0.1 U/mL, 1 U/mL, or 10 U/mL unfractionated heparin, or saline as a control. As measured by ELISA, a significant increase in media sFlt-1 was observed in groups treated with 0.1 U/mL (163.4 ± 12.9 pg/mL, *p* = 0.0001), 1 U/mL (332.8 ± 18.3 pg/mL, *p* < 0.0001), and 10 U/mL (257.8 ± 35.7 pg/mL, p < 0.0001) unfractionated heparin compared to the saline-treated control group (all measurements below detection), as shown in Fig. 1. There was also a significantly higher level of sFlt-1 in the media of BeWos treated with 1 U/mL (*p* < 0.0001) and 10 U/mL (*p* = 0.0230) unfractionated heparin compared to those treated with 0.1 U/mL unfractionated heparin. There was no difference between the media of BeWos treated with 1 U/mL and 10 U/mL unfractionated heparin.

**Fig. 2:**
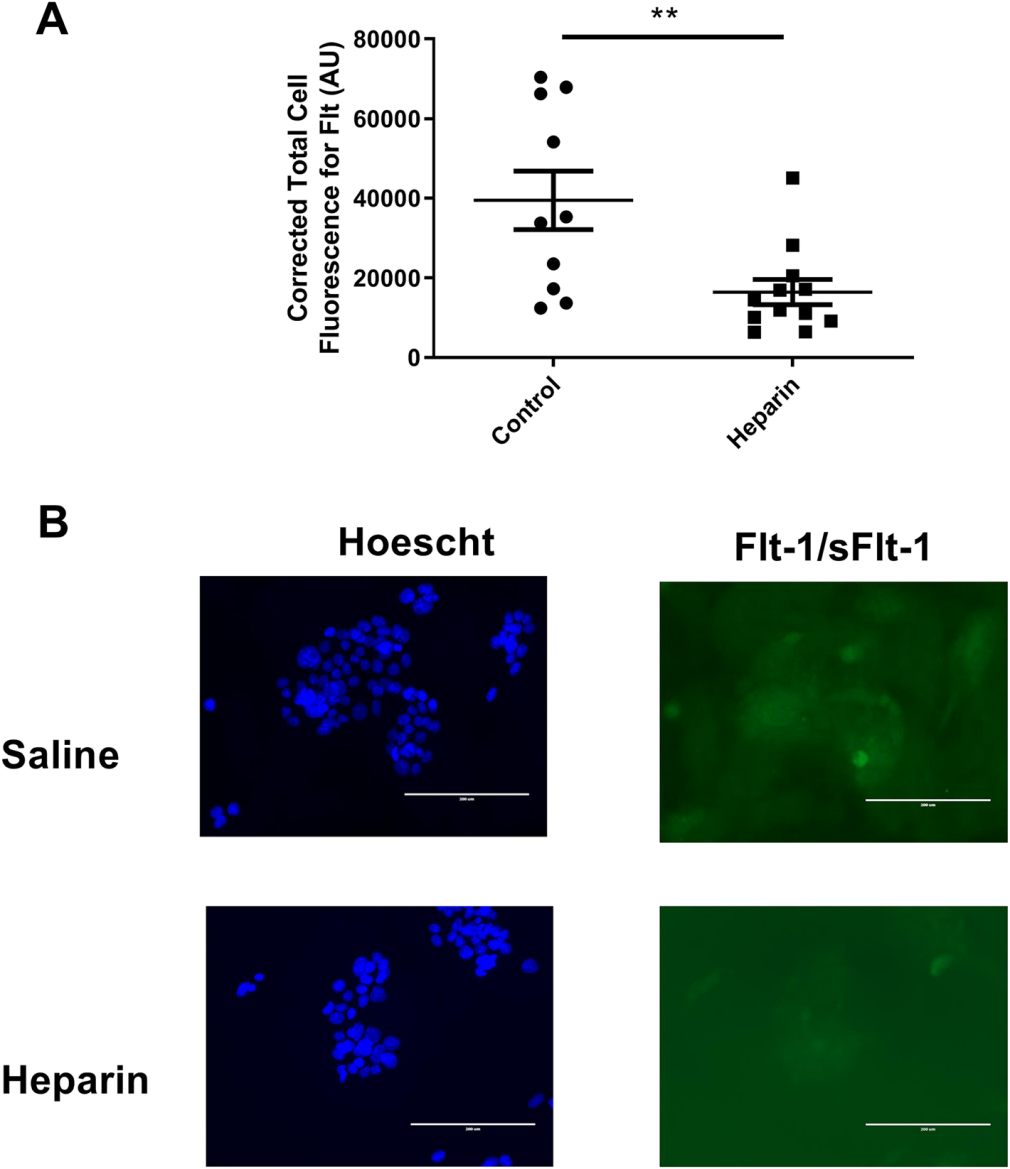
Flt fluorescence. BeWos cultured on 4-well chamber slides with or without 10 U/mL unfractionated heparin were probed for Flt (which includes both Flt-1 and sFlt-1) using a fluorescent antibody and visualized using an EVOS. Quantification of average fluorescence (**a**) and representative images (**b**) are presented. Hoescht, blue channel, indicates nuclei, while sFlt-1 fluorescence is shown in green channel. Scale bar represents 200 microns. *N* = 10–12 per group. Statistical analysis performed using unpaired Student’s *t* test. Significance is designated by a bar connecting groups (*p < 0.05, **p < 0.01, ***p < 0.001)

To determine whether the increased media sFlt-1 seen with our unfractionated heparin treatment was from additional sFlt-1 synthesis or shedding of existing sFlt-1, we measured the total cell immunofluorescence for Flt (this includes full-length Flt-1 receptors and matrix-bound sFlt-1) in unfractionated heparin-treated and vehicle treated BeWos. Interestingly, we found that the total cell fluorescence for Flt was significantly lower in the heparin-treated group compared to the control group (16461 ± 3169 AU vs. 39476 ± 7367 AU, respectively; *p* = 0.0063), as shown in Fig. 2.

### Heparin displaces sFlt-1 from placental explants ex vivo

**Fig. 3:**
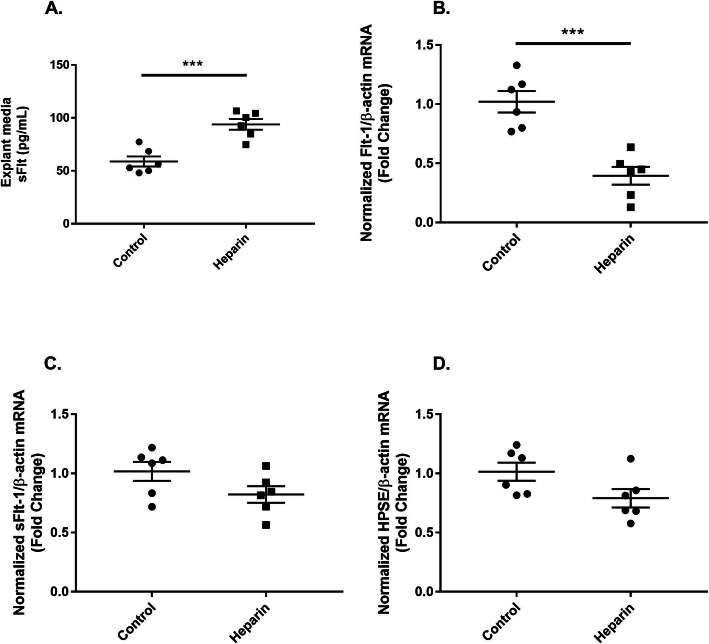
Heparin displaces sFlt-1 from placental explants. sFlt-1 protein levels in the media of placental explants cultured with saline or unfractionated heparin (10 U/mL) for 30 min, as measured by ELISA (**a**). qRT-PCR measurements of the expression of Flt-1 (**b**), sFlt-1 (**c**), and heparanase (HPSE) (**d**), all of which have been normalized to β-actin expression. *N* = 6 for all groups. Statistical analysis performed using unpaired Student’s *t* test. Significance is designated by a bar connecting groups (*p < 0.05, **p < 0.01, ***p < 0.001)

Looking to replicate our findings in an ex vivo model, we next treated placental explants, which were harvested from normal pregnant rats, with unfractionated heparin or saline vehicle as a control. As measured by ELISA, media from the unfractionated heparin-treated group had significantly higher sFlt-1 levels compared to media from controls (93.86 ± 5.06 pg/mL vs. 58.83 ± 4.72 pg/mL, respectively; *p* = 0.0005), as shown in Fig. 3a. We observed decreased expression of Flt-1 mRNA levels in the unfractionated heparin-treated explants compared to controls, as measured by qRT-PCR (0.39 ± 0.08 fold change normalized to β-actin vs. 1.02 ± 0.09 fold change normalized to β-actin , respectively; *p* = 0.003) (Fig. 3b). There was no difference in sFlt-1 expression between treated and untreated groups (Fig. 3c). Expression of heparanase-1, the predominant cleaving enzyme for heparan sulfate, was also not altered between groups (Fig. 3d).

### Chronic heparin administration modulates circulating sFlt-1 and maternal blood pressure in vivo

**Fig. 4:**
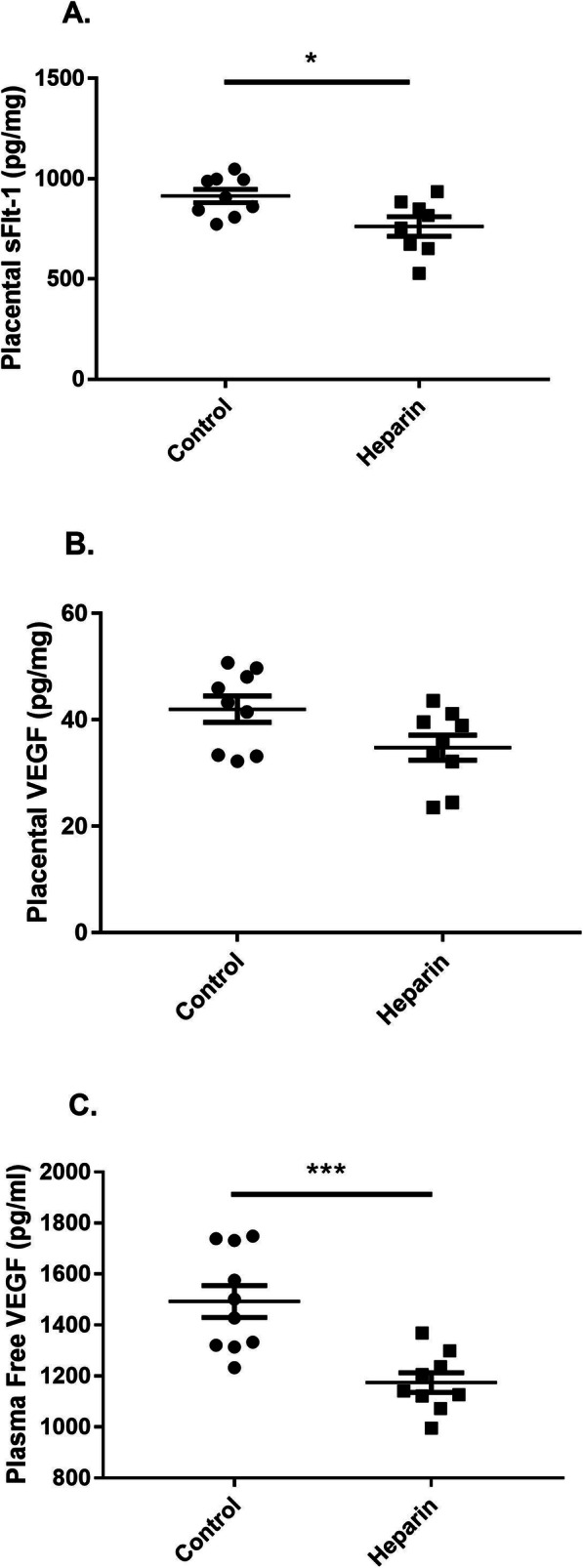
Heparin alters sFlt-1 and VEGF localization. Placental sFlt-1 protein levels in pregnant Sprague Dawley rats infused with either unfractionated heparin (100 U/kg/day) or saline as a control, as measured by ELISA (**a**). VEGF protein levels in the placenta (**b**) and plasma (**c**), as measured by ELISA. Placental measurements expressed as pg of sFlt-1 or VEGF per milligram of placental protein. *N* = 9–10 per group. Statistical analysis performed using unpaired Student’s *t* test. Significance is designated by a bar connecting groups (*p < 0.05, **p < 0.01, ***p < 0.001)

**Fig. 5:**
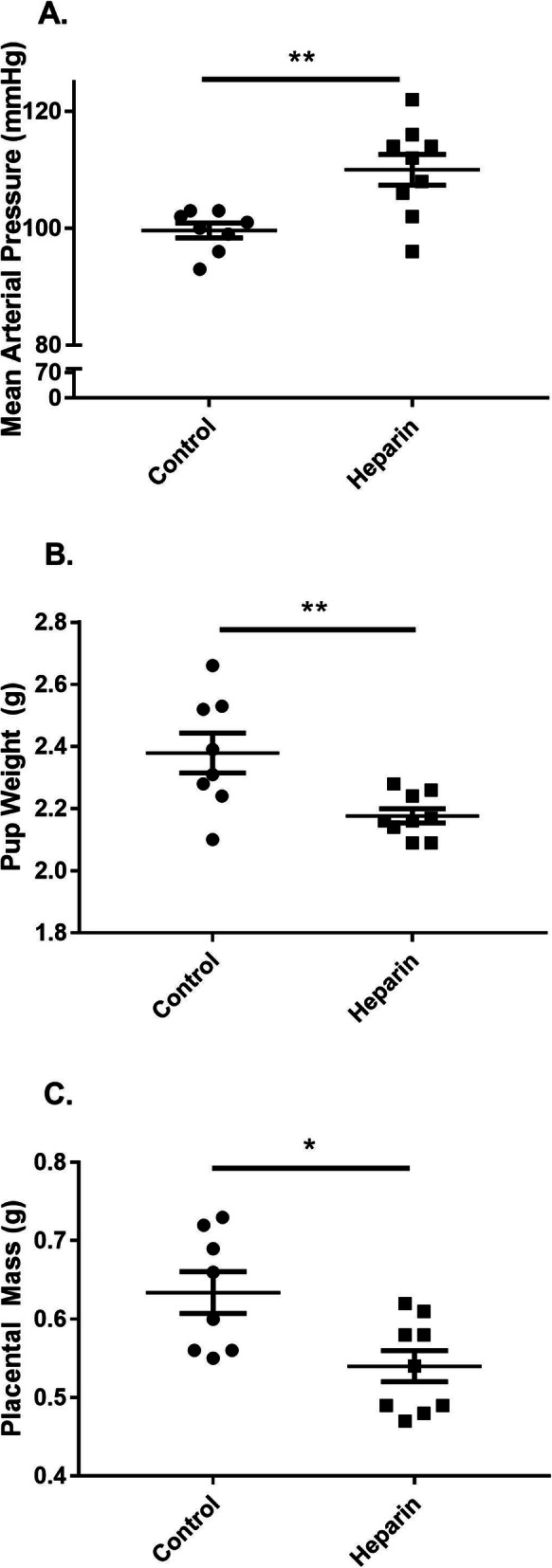
Heparin alters maternal blood pressure and offspring weight. Mean arterial pressure of pregnant Sprague Dawley rats infused with either unfractionated heparin (100 U/kg/day) or saline as a control, measured in conscious animals on gestational day 19 via indwelling carotid catheter (**a**). Average pup weight on gestational day 19 (**b**). Average placental mass on gestational day 19 (**c**). *N* = 9–10 per group. Statistical analysis performed using unpaired Student’s *t* test. Significance is designated by a bar connecting groups (*p < 0.05, **p < 0.01, ***p < 0.001)

Next, we moved to an animal model to determine whether heparin infusion displaced extracellular matrix-bound sFlt-1 in vivo. Pregnant Sprague Dawley rats infused with unfractionated heparin had significantly decreased placental sFlt-1 compared to saline-infused control animals as measured by ELISA (761.8 ± 48.39 pg/mg vs. 913.8 ± 32.56 pg/mg, respectively; *p* = 0.0178) (Fig. 4a). There was also a near-significant decrease in VEGF levels in placentas from treated animals compared to those from control animals (34.78 ± 2.36 pg/mg vs. 41.99 ± 2.47 pg/mg, respectively; *p* = 0.0505) (Fig. 4b) and a significant decrease in plasma free VEGF, which is VEGF unbound by sFlt-1 (1174 ± 38.45 pg/mL vs. 1492 ± 62.25 pg/mL, respectively; *p* = 0.0006) (Fig. 4c). These trends in the unfractionated heparin-treated rats were accompanied by a significant increase in mean arterial pressure compared to control animals (110 ± 2.63 mmHg vs. 99.63 ± 1.25 mmHg, respectively; *p* = 0.0038), as shown in Fig. 5a. Although the mechanism was not clear, unfractionated heparin treatment significantly reduced pup weight (2.38 ± 0.06 g in control rats vs. 2.18 ± 0.02 g in heparin-treated rats; *p* = 0.0075) and placental mass (0.63 ± 0.03 g in control rats vs. 0.54 ± 0.02 g in heparin-treated rats; *p* = 0.0116) (Fig. 5b, c, respectively).

## Discussion

sFlt-1 is a well-characterized anti-angiogenic molecule with the vital physiological role of limiting the activity of VEGF and other pro-angiogenic proteins [1, 18]. However, it has also been implicated in multiple diseases with underlying angiogenic imbalance, especially those with evident endothelial dysfunction such as cardiovascular disease, chronic kidney disease, and preeclampsia [6, 19, 20]. The importance sFlt-1 levels play in angiogenic balance and endothelial function is further illustrated by its ability to predict outcomes and disease severity in multiple diseases [19, 21–23]. Despite these associations and indications that sFlt-1 may serve as a biomarker for diseases with endothelial dysfunction, the origin of excess sFlt-1 in many diseases remains unknown.

While it may seem counterintuitive, many cell types other than endothelial cells produce and secrete sFlt-1. One cell type of interest to our group is the placental trophoblast, which is believed to contribute to the high level of sFlt-1 seen during pregnancy [24]. It is not by coincidence that the trophoblasts of the placenta, specifically syncytiotrophoblasts which interface with maternal circulation, also possess a mechanism for storing and buffering sFlt-1 in order to regulate its local and circulating levels. The ECM of syncytiotrophoblasts contains an abundance of heparan sulfate [9] just as the endothelial ECM does [25, 26], making the placenta capable of localizing and storing sFlt-1 through its molecular interactions with heparan sulfate [7].

Because the placental trophoblastic layer acts as a reservoir of sFlt-1, it would reason that dysfunction or manipulation of this system could release excessive amounts of sFlt-1 into the maternal circulation. Here, we have used exogenous heparin to determine the physiological role of the buffering mechanism of the placental ECM in sFlt-1 regulation during pregnancy. This is, in fact, what our results suggest, as cultured BeWo cells and placental villous explants readily mobilized reserve sFlt-1 upon exposure to heparin. We chose to use BeWos for our study because they share many characteristics with placental syncytiotrophoblasts [27], which are likely the largest source of sFlt-1 in the placenta. Moreover, because sFlt-1 can bind unfractionated heparin [8] and heparan sulfate [7], unfractionated heparin was ideal to probe the ECM of trophoblasts for sFlt-1 reservoirs. It should be mentioned that separate studies have proven that unfractionated heparin and low-molecular-weight-heparin can both increase circulating sFlt-1 [28, 29]. Currently, differences in the affinities that the two types of heparin possess for sFlt-1, if there are any at all, are not known.

We observed that both BeWos and placental villous explants release sFlt-1 from their ECM into their culture media upon treatment with unfractionated heparin, which we believe is actively competing with heparan sulfate for sFlt-1 binding. This notion is further supported by our immunofluorescence staining of BeWos. In conjunction with increased sFlt-1 release, we found a decrease in localized Flt fluorescence on BeWo cell surfaces after treatment, presumably due to the loss of sFlt-1 from the surface of the cells’ ECM, and not increased production. Additionally, upon measuring the expression of sFlt-1 in heparin-treated placental villous explants, we found no increase in sFlt-1 mRNA, further supporting the notion that preexisting sFlt-1 was displaced from the ECM. In fact, we observed a decrease in production of Flt-1, the membrane bound variant of sFlt-1. It is possible that Flt-1 downregulation was an attempt to re-establish angiogenic balance through a negative feedback mechanism after the release of large quantities of sFlt-1. To determine if sFlt-1 release from the ECM was due to competitive binding of unfractionated heparin or enzymatic cleavage of heparan sulfate chains with bound sFlt-1, we measure the expression of the extracellular matrix enzyme heparanase. Expression of heparanase-1, the predominant enzymatic cleaver of heparan sulfate, was unaltered in placental villous explants subjected to unfractionated heparin. The confirmation that heparanase-1 was not upregulated by treatment with unfractionated heparin supports our conclusion that sFlt-1 is being released from the ECM as a consequence of competition for binding between unfractionated heparin and heparan sulfate.

While this data is highly suggestive, replication of our results was needed in pregnant animals to conclude that placental heparan sulfate-bound sFlt-1 is present in large quantities and can be mobilized to a significant enough degree to raise blood pressure. In our study, pregnant Sprague Dawley rats infused with unfractionated heparin had significant reductions in localized placental sFlt-1. We believe that unfractionated heparin competed with the heparan sulfate present on the surface of placental syncytiotrophoblasts for sFlt-1 binding. Because the placental syncytiotrophoblast layer lies in direct contact with the maternal circulation and bathes in maternal blood, sFlt-1 can be carried away from the placenta and distributed throughout the maternal circulation. While we could not measure circulating sFlt-1 in these animals (due to technical limitations), previous clinical studies have showed that heparin administration leads to increased circulating sFlt-1 in humans [10, 29–31]. Placental VEGF also trended towards reduction (*p* = 0.0505), likely because VEGF contains a heparan sulfate-binding domain which can also bind heparin [32], much in the same way sFlt-1 can bind either of these molecules. Therefore, it reasons that unfractionated heparin is competing with heparan sulfate for sFlt-1, and possibly VEGF, binding and modulating localizations. Despite any potential mobilization of placental heparan sulfate-bound VEGF, plasma levels of free bioavailable VEGF (unbound by sFlt-1) were reduced. A probable explanation is the large amount of sFlt-1 that was released from the placental ECM during treatment bound to, and sequestered, circulating free VEGF. From here, it follows that the presence of excess sFlt-1 could cause angiogenic imbalance and then systemic endothelial dysfunction, which contributes to the development of clinical manifestations, including hypertension. Supporting this, we observed an increase in blood pressure in our unfractionated heparin-infused group, although it appeared to be subclinical. Previous studies by other research groups have observed the effects of heparin administration on sFlt-1 levels in human patients with coronary artery disease [30], undergoing dialysis [31], and in pregnancy and preeclampsia [10, 29]. However, only one such study, by Rosenberg et al. [10] in non-complicated pregnancies, measured blood pressure, and they found no differences between women given heparin and those who did not. To our knowledge, the current study is the first to present data indicating that sFlt-1 can be mobilized from the placenta by unfractionated heparin and that this mobilization can be significant enough to affect maternal blood pressure. Interestingly, we also observed reduced offspring weight in the heparin-treated animals. Although the mechanism was not entirely clear, it is possible that excessive mobilized sFlt-1 antagonized angiogenesis in the blood vessels supporting the placentas, thus preventing those vessels from fully remodeling. This would have impaired their ability to support the growing fetuses’ nutritive demand and left the offspring growth restricted. Thus, through our probing with unfractionated heparin, we have revealed that sFlt-1 is housed in the placental ECM in vast quantities during pregnancy and that mobilization of sFlt-1 by any means can cause angiogenic imbalance and clinical manifestations of disease.

Consequently, the most obvious application of our findings is in the field of preeclampsia, which we have described as being a disease of angiogenic imbalance, endothelial dysfunction, and hypertension. It is well characterized that the placenta is ischemic and dysfunctional in preeclampsia and in experimental models of preeclampsia [11, 33–36]. Several genes are differentially expressed in the ischemic placenta, which contribute greatly to the disorder [37]. In fact, our group has recently shown that expression of heparanase, an enzymatic cleaver of heparan sulfate, was upregulated in the trophoblast-like BeWo cell line during hypoxia and that this leads to increased sFlt-1 release [13]. Furthermore, inhibiting heparanase attenuated the hypoxia-induced sFlt-1 release from BeWos. Taken together, these studies illustrate a process whereby an ischemic placenta could upregulate heparanase, increase heparan sulfate cleavage, and release vast quantities of previously bound sFlt-1, leading to hypertension and other manifestations. The current study indicates that the placental ECM is a large storage site of sFlt-1 and that its disruption and release by any means, here through the use of competitive binding with unfractionated heparin, can cause maternal manifestations of disease.

## Perspectives and significance

While previous studies have demonstrated that infusion of heparin could modulate circulating sFlt-1 levels in both non-pregnant and pregnant individuals, the source of the released sFlt-1 and the physiological effects of its release under these conditions were not readily clear. Here, we have directly measured the effects of chronic heparin administration during pregnancy and demonstrated sFlt-1 release from placental cells and tissues, altered maternal angiogenic balance, and sub-clinical hypertension as a result of this treatment. Additionally, our data suggests that ECM-bound sFlt-1 can be mobilized into the circulation to a significant enough degree to manifest clinical aspects of angiogenic imbalance and endothelial dysfunction during pregnancy. Again, our group has recently shown that heparanase, a proteolytic enzyme that cleaves and solubilizes heparan sulfate, is upregulated during hypoxia and that this increases sFlt-1 release in the BeWo cell line. Our previous data demonstrating the role of heparanase in hypoxia along with the current study establishing the placenta as a large sFlt-1 reservoir suggest that ECM cleavage and subsequent solubilization of previously bound sFlt-1 may be a source for increased circulating sFlt-1 in disease. Future studies should aim to evaluate the significance of heparanase and other likely mechanisms that could lead to the cleavage of heparan sulfate and release of ECM-bound sFlt-1 in pregnancy-specific diseases of angiogenic imbalance and excess sFlt-1.

## Data Availability

The datasets used during the current study are available from the corresponding author upon reasonable request.
